# Silencing of PRDX2 Inhibits the Proliferation and Invasion of Non-Small Cell Lung Cancer Cells

**DOI:** 10.1155/2020/1276328

**Published:** 2020-04-03

**Authors:** Xu Jing, Lutao Du, Aijun Niu, Yunshan Wang, Yuli Wang, Chuanxin Wang

**Affiliations:** Department of Clinical Laboratory, The Second Hospital of Shandong University, Jinan 250033, China

## Abstract

Peroxiredoxin 2 (PRDX2), a member of the peroxiredoxin family of antioxidant enzymes, has been revealed to be an important player in cancer progression. However, the biological role of PRDX2 in the progression of non-small cell lung cancer (NSCLC) is poor reported. In the present study, the loss-of-function experiments were performed to investigate the specific role of PRDX2 in the growth and invasion of NSCLC. The results revealed that knockdown of PRDX2 by siRNA interference significantly suppressed the proliferation, migration, and invasion of A549 and H1299 cells, as well as diminished the activity of MMP9. Additionally, the decrease in PRDX2 expression significantly promoted apoptosis in NSCLC cells by downregulating expression of Bcl-2 and upregulating the expression of Bax, cleaved caspase 3 and cleaved caspase 9, but had no significant effect on the apoptosis of normal lung epithelial cells BEAS-2B. Moreover, PRDX2 inhibitor also inhibited the proliferation, migration, and invasion of A549 cells and promoted apoptosis. Further, our data demonstrated that silencing of PRDX2 markedly reduced the phosphorylation of Akt and mTOR and expression of downstream proteins Cyclin D1 and p70S6k. In conclusion, our findings indicate that PRDX2 exerts a prooncogenic role in the progression of NSCLC and might be a potential therapeutic target for NSCLC treatment.

## 1. Introduction

Lung cancer is the leading cause of cancer-related death in the world, with an estimated 1.8 million new cases diagnosed and 1.6 million deaths every year [1, 2]. More than 85% of the cases are non-small cell lung cancer (NSCLC), the commonest type of lung cancer, of which lung squamous cell carcinoma and lung adenocarcinoma are the most common subtypes [2–4]. Despite the important progress in diagnosis and treatment of NSCLC over the past two decades, the 5-year survival rate of NSCLC patients remains as low as 15% [4]. Local recurrence, metastasis, and drug resistance are important factors affecting the prognosis of NSCLC patients [5–7]. It is reported that approximately 79% of NSCLC patients develop subsequent metastasis, such as brain metastasis, and even metastatic disease has occurred at the time of diagnosis [8–10]. Therefore, it is necessary to further explore the molecular mechanism of NSCLC and identify novel therapeutic targets for the treatment of NSCLC.

Peroxiredoxin (PRDX), a family of thiol-specific antioxidant enzymes, is revealed to function in scavenging H_2_O_2_, alkyl hydroperoxide, and peroxynitrite and known as important player in a wide range of physiological and pathological processes [11]. Previous studies have shown that one of the known mechanisms of carcinogenesis is the activation of various signaling pathways and changes of transcription factors caused by prolonged oxidative stress [12, 13]. As a key member of PRDXs family, peroxiredoxin 2 (PRDX2) has been reported to scavenge peroxides and reactive oxygen species (ROS) in cells to regulate redox status, thus participating in a variety of cellular biological functions [14–16]. Recent studies identify aberrant expression of PRDX2 in several types of cancers and demonstrate that PRDX2 exerts an important role in cell proliferation, death, and drug sensitivity of cancer [17–20]. For example, it has been indicated that PRDX2 is upregulated in colorectal cancer and exerts a tumor promoting role in the progression of colorectal cancer [21, 22]. Using bioinformatics analysis, Chen et al. report that the expression of PRDX2 mRNA is associated with the overall survival in lung cancer patients; the high expression is correlated with worse overall survival of patients [23]. However, the biological role of PRDX2 in the progression of NSCLC has not yet been reported.

In this study, we aimed to investigate the specific role of PRDX2 in the growth and invasion of NSCLC. Our data demonstrated that knockdown of PRDX2 significantly inhibited the proliferation, migration, and invasion of NSCLC cells, as well as promoted apoptosis. In addition, silencing of PRDX2 reduced activation of the Akt/mTOR signaling pathway in NSCLC cells. All in all, these results indicated an oncogenic role of PRDX2 in the progression of NSCLC, and PRDX2 may function as a potential target for therapy of NSCLC.

## 2. Materials and Methods

### 2.1. Cell Culture and Transfection

Human normal lung epithelial cells BEAS-2B and the human NSCLC cell lines A549 and H1299, obtained from the cell bank of Chinese Academy of Sciences (Shanghai, China), were cultured in DMEM medium (Hyclone, Logan, Utah, USA) supplemented with 10% FBS (Gibco, Thermo Fisher Scientific, USA), 100 U/ml penicillin (Sigma, Louis, MO, USA) and 100 mg/ml streptomycin (Sigma) at 37°C with 5% CO_2_. After reaching 80% confluence, A549 and H1299 cells were transfected with siRNA against PRDX2, which was synthesized by RiboBio (Guangzhou, China) using Lipofectamine 2000 (Invitrogen, Carlsbad, CA, USA) according to the instructions, and siR-Ribo™ Negative Control (RiboBio) was used as negative control (si-NC). PRDX2 inhibitor was obtained from RiboBio and transfected into A549 cells using Lipofectamine 2000.

### 2.2. Quantitative Real-Time Polymerase Chain Reaction (qRT-PCR)

Total RNA was extracted from NSCLC cells transfected with siRNAs using an Ultrapure RNA Kit (CWBIO, Beijing, China) and reverse transcripted into cDNA by the HiFiScript cDNA Synthesis Kit (CWBIO). The PCR reaction was performed with FastSYBR Mixture (CWBIO) on an ABI 7500 Fast system (Applied Biosystems, Foster, CA). The following primers were used in this study: PRDX2, 5′-CCTTCAAAGAGGTGAAGCTG-3′ (forward) and 5′-GTTGCTGAACGCGATGAT-3′ (reverse); *β*-actin was used as the internal control, 5′-CCCGAGCCGTGTTTCCT-3′ (forward) and 5′-GTCCCAGTTGGTGACGATGC-3′ (reverse). The 2^-*Δ*ΔCt^ method was used to calculate the relative expression of PRDX2.

### 2.3. Western Blot Analysis

Following 48 h of transfection with siRNAs, the 6-well plate was placed on ice, and cells were lysed in RIPA lysate (CWBIO). The protein concentration was determined using a BCA Protein Assay Kit (CWBIO). 20 *μ*g of protein from each sample was separated by 10% SDS PAGE gel and electrotransferred onto a polyvinylidene fluoride membrane (PVDF; Millipore, Billerica, MA, USA) following to blocking with 5% nonfat milk for 1 h. After incubating with primary antibodies at 4°C overnight, the membrane was incubated with corresponding HRP-conjugated secondary antibodies (diluted 1 : 3000; cat.no. SA00001-1, 2; Proteintech, USA). Following being visualized with ECL kit (Millipore), the protein bands were quantified using the Image Lab software (Bio-Rad, USA). The primary antibodies used in this study were as follows: anti-PRDX2 (diluted 1 : 1000; cat.no. 10545-2-AP), anti-Bcl-2 (diluted 1 : 1000; cat.no. 12789-1-AP), anti-Bax (diluted 1 : 1000; cat.no. 50599-2-Ig), anti-Akt (diluted 1 : 1000; cat.no. 10176-2-AP), anti-p-Akt (diluted 1 : 1000; cat.no. 66444-1-Ig), anti-mTOR (diluted 1 : 1000; cat.no. 20657-1-AP), anti-MMP9 (diluted 1 : 1000; cat.no.10375-2-AP), and GAPDH (diluted 1 : 1000; cat.no. 60004-1-Ig) were obtained from Proteintech; anti-cleaved caspase 3 (diluted 1 : 1000; cat.no. 9661), anti-cleaved caspase 9 (diluted 1 : 1000; cat.no. 9505), and anti-p-mTOR (diluted 1 : 1000; cat.no. 2971) were obtained from Cell Signaling Technology (Danvers, MA, USA).

### 2.4. CCK8 Assay

NSCLC cells transfected with siRNAs for 24 h were seeded in a 96-well plate at a density of 3,000 cells per well and incubated at 37°C for different periods (0, 24, 48, and 72 h). CCK8 regent (10 *μ*l/well; Solarbio, Beijing, China) was added each well and incubated at 37°C for 1 h before the absorbance was measured at 450 nm.

### 2.5. Colony Formation Assay

After 24 h of transfection, the cells were digested with 0.25% trypsin and prepared cell suspension. Cells were plated in 35 mm dishes containing 5 ml medium at a concentration of 300 cell/dish. Following incubation at 37°C for 1-2 weeks, cells were washed with PBS and fixed with 4% polyformaldehyde for 30 min. Then, colonies were stained with 0.1% crystal violet for 30 min, then slowly washed with running water and air dried. The colonies were counted and captured under a light microscope.

### 2.6. Wound-Healing Assay

After 24 h of transfection, cells were seeded in a 6-well plate. When the cell density reached 100% confluence, a scratch was generated using a 200 *μ*l pipette tip and washed with PBS to remove the scratched cells. Following adding medium, cells were cultured at 37°C for another 24 h. Cells were captured using a light microscope, and ImageJ software was used to analyze the percentage of wound closure.

### 2.7. Transwell Assay

Transwell chambers (Millipore) precoated with or without Matrigel were performed for cell invasion or migration assay, respectively. NSCLC cells after 24 h of transfection were collected and suspended in serum-free medium; 100 *μ*l of cell suspension (1 × 10^5^ cells) was added to the upper chamber, and 500 *μ*l of the DMEM medium containing 10% FBS was added to the lower chamber. After culturing at 37°C for 24 h, the residual cells in the upper chamber were wiped off with a cotton swab, and the migrated or invaded cells were fixed with 4% paraformaldehyde for 30 min and stained with 0.1% crystal violet for 20 min. Five fields of view were randomly selected under the microscope to count and photograph the migrated or invaded cells (magnification, x100).

### 2.8. Gelatin Zymography Analysis

A549 and H1299 cells transfected with siRNAs for 24 h were washed three times with serum-free DMEM and then cultured at 37°C for another 24 h with serum-free DMEM. The supernatants were collected and electrophoresed by 10% SDS-PAGE gel containing with 0.5 mg/ml gelatin. After electrophoresis, the gel was eluted and stained in 0.25% Coomassie Brilliant Blue R-250 for 4 h at room temperature. Following decolorization at room temperature, the gel was scanned by the Image Scanner (Ahmad Sohm, USA) and analyzed by IMAGISOANT TL V2003 software.

### 2.9. Hoechst33342/PI Staining Assay

Following transfection with siRNAs for 24 h, NSCLC cells were harvested and dual-stained with Hoechst33342 (10 *μ*l; Beyotime, Shanghai, China) at 37°C for 5-15 min and PI (5 *μ*l; Beyotime) in the dark at room temperature for 10 min. Cells were captured and counted with a fluorescent microscope.

### 2.10. Flow Cytometry Analysis

Cells were cultured in a serum-free medium for 24 h and stained with the Annexin V-FITC/PI Apoptosis Detection Kit (CWBIO). Then, cells were subjected to apoptosis analysis using a flow cytometer (BD FACSCanto II, BD Biosciences, USA).

### 2.11. Statistical Analysis

Data were presented as means ± SD of at least three independent experiments. GraphPad software 7.0 (GraphPad Inc., USA) was used for the statistical analysis in this study. The Student's *t* test was performed to analyze differences between two groups, and one-way ANOVA was used for comparison between 3 groups or more. *P* < 0.05 indicated a statistically significant difference.

## 3. Results

### 3.1. Knockdown of PRDX2 Inhibits the Proliferation of NSCLC Cells

**Figure 1:**
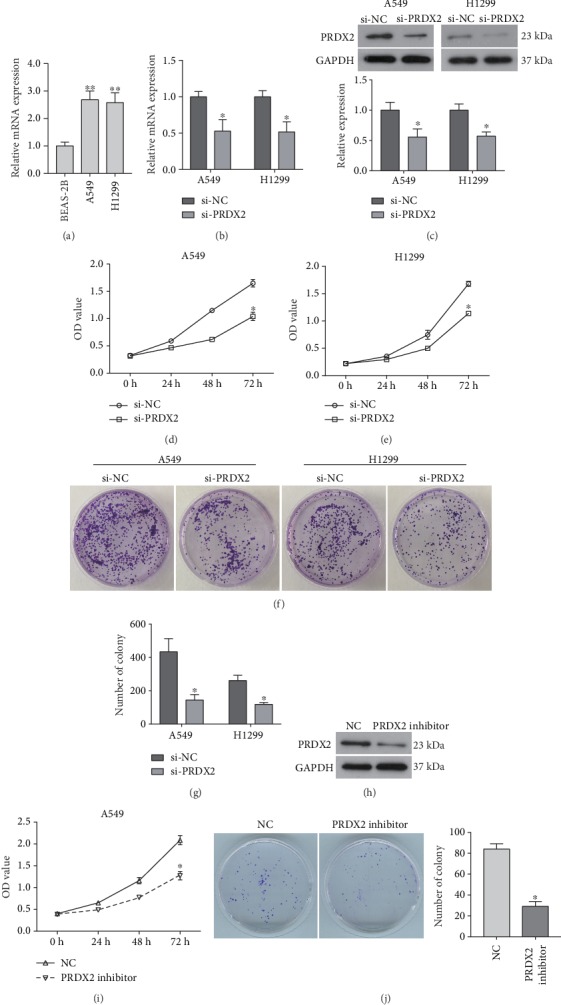
Knockdown of PRDX2 inhibits the proliferation of NSCLC cells in vitro. (a) Expression of PRDX2 mRNA in BEAS-2B, A549, and H1299 cells was determined using qRT-PCR assay. A549 and H1299 cells were transfected with siRNA-PRDX2 (si-PRDX2), and siRNA negative control was used as negative control (si-NC). (b) Following transfection of 24 h, the expression of PRDX2 mRNA was determined using qRT-PCR assay. (c) The relative expression of PRDX2 protein in NSCLC cells transfected with siRNAs for 48 h was examined using western blot analysis. (d, e) CCK8 assay was performed the viability of A549 (d) and H1299 (e) cells. (f) Colony formation assay was used to assess cell proliferation. (g) Quantitative analysis of the results of colony formation assay. (h) The expression of PRDX2 protein in A549 cells transfected with PRDX2 inhibitor was examined using western blot analysis. (i) CCK8 assay was performed to measure A549 cell viability after indicated treatment. (j) Colony formation assay was used to assess A549 cell proliferation. ^∗^*P* < 0.05, ^∗∗^*P* < 0.01.

As indicated by qRT-PCR analysis, we found that the expression of PRDX2 mRNA was markedly upregulated in NSCLC cell lines A549 and H1299 compared to normal lung epithelial cells BEAS-2B (Figure 1(a)). Therefore, to investigate the biological role of PRDX2 in the progression of NSCLC, we silenced its expression using siRNA-PRDX2 interference in A549 and H1299. As shown in Figures 1(b) and 1(c), siRNA-PRDX2 significantly inhibited the expression of PRDX2 at both mRNA and protein levels. CCK8 assay showed that silencing of PRDX2 inhibited the viability of A549 cells as compared with NC group (Figure 1(d)). Similarly, a significant decrease in the viability of H1299 cells transfected with siRNA-PRDX2 was also observed (Figure 1(e)). Consistent with these results, siRNA-PRDX2 also reduced the colony formation abilities of A549 and H1299 cells compared with the control cells (Figures 1(f) and 1(g)). Additionally, downregulation of PRDX2 by PRDX2 inhibitor also significantly inhibited the viability and colony formation ability of A549 cells compared with the corresponding control group (Figures 1(h)–1(j)). These results suggest that loss of PRDX2 suppresses the proliferation of NSCLC cells in vitro.

### 3.2. Silencing of PRDX2 Suppresses the Migration and Invasion of NSCLC Cells

**Figure 2:**
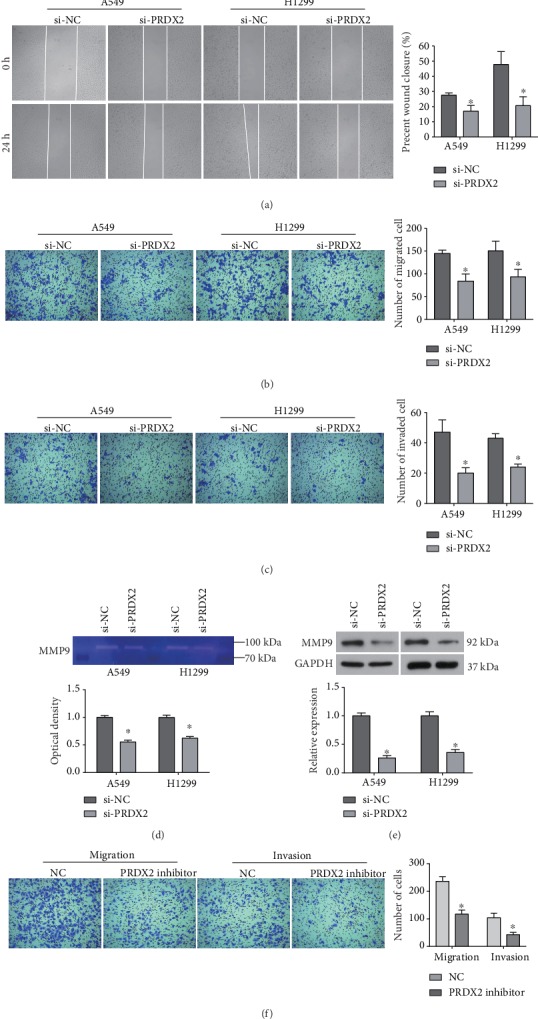
Silencing of PRDX2 suppresses the migration and invasion of NSCLC cells. (a) Cell migration in A549 and H1299 cells transfected with siRNAs was assessed using wound-healing assay. (b, c) Transwell assay was performed to determine cell migration (b) and invasion (c) in cells transfected with siRNAs for 24 h. (d) The MMP9 activity was examined in A549 and H1299 cells transfected with siRNAs. (e) Expression of MMP9 protein was detected by western blot analysis. (f) A549 cells migration and invasion were measured by transwell assay. ^∗^*P* < 0.05.

A wound-healing assay was performed to evaluate the effect of PRDX2 on the metastasis capability of NSCLC cells. As indicated in Figure 2(a), knockdown of PRDX2 significantly inhibited the ability of A549 and H1299 cells to migrate into the blank space. Similar to above, a transwell assay also revealed a significant decrease in cell migration caused by siRNA-PRDX2 in both A549 and H1299 cells (Figure 2(b)). Moreover, A549 and H1299 cells transfected with siRNA-PRDX2 displayed a significant depression in the invasion ability compared with control cells (Figure 2(c)).

Considering the key role of MMP9 in cell adhesion and invasion [24], we examined the activity of MMP9 using gelatin zymography analysis. As shown in Figure 2(d), knockdown of PRDX2 significantly diminished the activity of MMP9 in both A549 and H1299 cells. The expression of MMP9 protein was also significantly downregulated by knockdown of PRDX2 (Figure 2(e)). Further, PRDX2 inhibitor also suppressed A549 cell migration and invasion (Figure 2(f)). Collectively, our data indicate that loss of PRDX2 may suppress the migration and invasion abilities of NSCLC cells by downregulating the activity of MMP9.

### 3.3. Knockdown of PRDX2 Promotes the Apoptosis of NSCLC Cells

**Figure 3:**
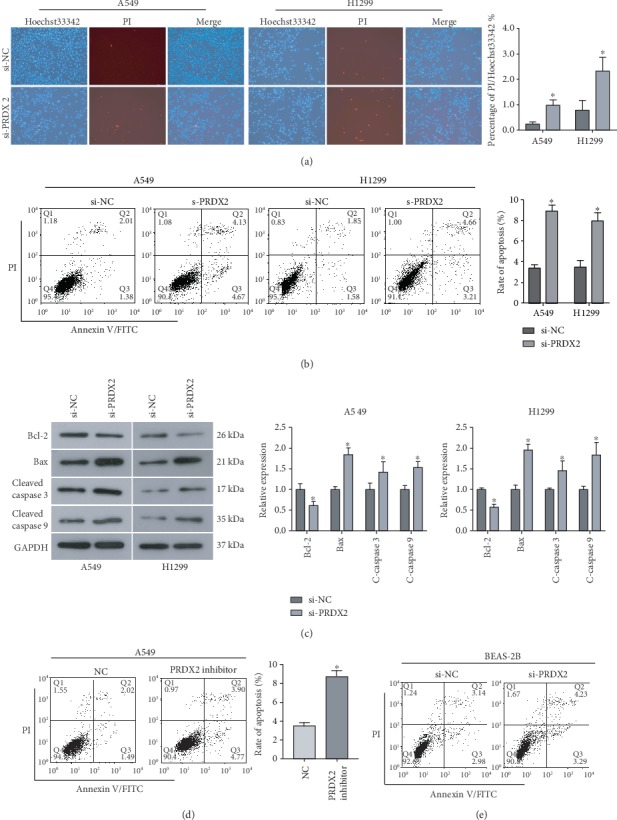
Knockdown of PRDX2 promotes cell apoptosis by regulating the Bcl-2/Bax axis and caspase cascade. (a) Hoechst33342/PI staining assay was used to assess apoptosis in A549 and H1299 cells when PRDX2 was silenced. (b) Flow cytometry analysis was performed to measure the percentage of apoptotic cells in A549 and H1299 cells after indicated treatment. (c) The expression of apoptosis-related proteins, Bcl-2, Bax, cleaved caspase 9, and cleaved caspase 9, was examined using western blot analysis. (d) Apoptosis in A549 cells transfected with PRDX2 inhibitor was measured by flow cytometry analysis. (e) Apoptosis in BEAS-2B cells transfected with si-PRDX2 was measured by flow cytometry analysis. ^∗^*P* < 0.05.

It is generally accepted that avoiding death is a major feature of tumor cells. Thus, we further investigated the effect of PRDX2 on apoptosis in NSCLC. As demonstrated in Figure 3(a), silencing of PRDX2 could promote apoptosis of NSCLC cells, the apoptotic rate of PRDX2-knockdown cells was significantly higher than the NC group. Flow cytometry results further confirmed that silencing PRDX2 increased apoptotic rate of both A549 and H1299 cells compared with corresponding control group (Figure 3(b)). Therefore, further study was conducted to investigate the mechanism underlying the induced apoptosis by siRNA-PRDX2 in NSCLC cells. Our data revealed that silencing of PRDX2 significantly downregulated the expression of antiapoptotic protein Bcl-2, while upregulated the expression of proapoptotic protein Bax in both A549 and H1299 cells (Figure 3(c)). Additionally, the activation of caspase 3 and caspase 9, which play crucial roles in apoptotic process, was both increased by siRNA-PRDX2 in A549 and H1299 cells (Figure 3(c)). In addition, our data indicated that PRDX2 inhibitor also promoted the percentage of apoptotic A549 cells (Figure 3(d)). However, silencing PRDX2 had no significant effect on the apoptosis of BEAS-2B (Figure 3(e)). Therefore, these results provide evidence that downregulation of PRDX2 promotes the apoptosis of NSCLC cells by regulating the Bcl-2/Bax axis and caspase cascade.

### 3.4. Silencing of PRDX2 Inhibits Activation of the Akt/mTOR Signaling Pathway in NSCLC Cells

**Figure 4:**
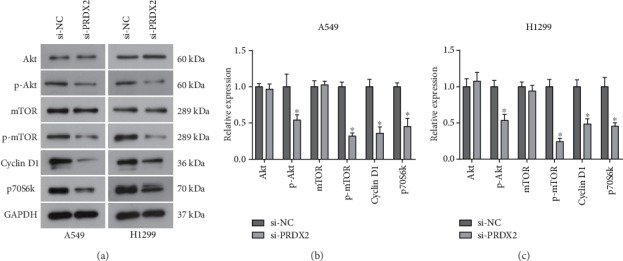
Knockdown of PRDX2 inhibits activation of the Akt/mTOR signaling pathway in NSCLC cells. (a) Following transfection of 48 h, the protein expression of Akt, p-Akt, mTOR, p-mTOR, Cyclin D1, and p70S6k in A549 and H1299 cells after si-PRDX2 or si-NC transfection. (b, c) Quantitative analysis of western blot results in A549 (b) and H1299 (c) cells. ^∗^*P* < 0.05.

It is well known that the Akt/mTOR signaling pathway plays important roles in cellular physiological processes, including cell proliferation, differentiation, invasion, and survival. The Akt/mTOR signaling pathway has been revealed to be overactivated in tumors and involved in tumor progression, including NSCLC [25, 26]. Herein, western blot analysis was performed to evaluate the effect of PRDX2 on the Akt/mTOR signaling pathway in NSCLC. As demonstrated in Figures 4(a)–4(c), silencing of PRDX2 significantly decreased the protein levels of phosphorylated Akt and mTOR in both A549 and H1299 cells, while the expression of total Akt or mTOR was not affected. Moreover, the expression of the downstream proteins Cyclin D1 and p70S6k, key regulators involved in cell proliferation, was correspondingly inhibited by siRNA-PRDX2 compared with the control cells (Figures 4(a)–4(c)). Taken together, our results indicate that targeting PRDX2 could inhibit activation of the Akt/mTOR signaling pathway in NSCLC cells.

## 4. Discussion

PRDXs has been reported to function in balancing cellular ROS and cytokine-induced peroxides and affect cell signaling transduction, regulating diversity biological behaviors, including tumorigenesis and cancer progression [27–29]. Emerging evidences reveal that PRDX2 functions a protumorigenic role in cancer progression. It has been identified that PRDX2 is overexpressed in various type of cancers including colorectal cancer [30] and cervical cancer [31] and associated with the tumor metastasis and prognosis of patients [22]. Knockdown of PRDX2 could inhibit the growth and promote apoptosis of colorectal cancer cells and increase the sensitivity of colon cancer cells to 5-FU [19, 21]. However, PRDX2 is considered to perform an inconsistent function in hepatocellular carcinoma (HCC). Bai B et al. reveal that PRDX2 is downregulated in HCC tissues and cells, silencing of PRDX2 significantly promotes the proliferation and migration of HCC cells, indicating a tumor-suppressing role of PRDX2 in HCC [32]. By contrast, Zhou S et al. show that decreasing of PRDX2 enhances H_2_O_2_-induced cell death in HCC SMMC-7721 cells, while overexpression of PRDX2 shows an opposite effect, indicating a protumorigenic role of PRDX2 in HCC [33]. Our study provided further evidence in support of the tumor-promoting role of PRDX2 in cancer progression. In the current study, we demonstrated that downregulation of PRDX2 significantly inhibited the proliferation, migration, and invasion abilities of NSCLC cells.

It is known that besides proliferation, apoptosis also plays a crucial role in the development of cancer, and dysregulated apoptosis is a major feature of tumor cells. PRDX2 has been confirmed to regulate tumor cell apoptosis [19]. Herein, we found that a decrease in PRDX2 expression resulted in an increase in apoptosis in NSCLC cells, but had no significant effect on apoptosis in BEAS-2B cells. Bcl-2 family is generally known to play essential roles in triggering apoptosis, and the Bcl-2/Bax is the most important regulator which can determine cell fate [34]. Additionally, activated caspase 9 is involved in the initiation of a series of apoptotic events, and the activated caspase 3 is the key executor of apoptosis. In the current study, we found that knockdown of PRDX2 downregulated the expression of Bcl-2, while upregulated the expression of Bax, cleaved caspase 3 and cleaved caspase 9 in NSCLC cells. Zhang S et al. report that silencing PRDX2 upregulated the expression of cleaved caspase 3, caspase 7, caspase 9, and Bad in gastric cancer cells [35]. Therefore, PRDX2 regulates apoptosis in NSCLC through regulating the Bcl-2/Bax axis and caspase cascade. All in all, these results suggest that PRDX2 exerts a prooncogenic role in the progression of NSCLC. Previous study reveals that PRDX2 nitrosation regulated by GSNO can cause intracellular H_2_O_2_ accumulation and induce lung cancer cell death [36]. However, it is unclear whether the oncogenic role of PRDX2 is associated with its antioxidant activity, and further study is required to investigate this.

As a crucial mechanism involved in cellular physiological processes and tumor progression, the Akt/mTOR signaling pathway represents a hotspot in the treatment strategy of cancer [37]. Activation of the Akt/mTOR signaling pathway is capable of regulating the expression of downstream effectors, such as Cyclin D1 and p70S6k, participating in cellular functions. More importantly, it has been revealed that PRDX2 regulates the resistance of colon cancer cells to 5-FU by regulating the Akt signaling pathway [19]. To determine whether PRDX2 impacts the Akt/mTOR signaling pathway in NSCLC cells, we examined the expression of important components of this signaling pathway in the siRNA-PRDX2 transfected cells. As indicated by western blot analysis, we observed that silencing of PRDX2 suppressed activation of the Akt/mTOR signaling pathway by decreasing the phosphorylation of Akt and mTOR and expression of Cyclin D1 and p70S6k. Collectively, the Akt/mTOR signaling pathway is involved in the tumor-promoting effect of PRDX2 in NSCLC cells. Ma Y et al. has showed that DNM3 upregulated by silencing PRDX2 inhibits the proliferation and promotes apoptosis in colon cancer cells by suppressing the Akt signaling pathway [38]. However, it remains unclear how PRDX2 regulates the Akt/mTOR signaling pathway, affecting NSCLC cell growth and apoptosis. Further analysis is required to investigate the PRDX2/Akt/mTOR signaling in our future study.

In summary, our findings revealed a prooncogenic role of PRDX2 in the progression of NSCLC, downregulation of PRDX2 significantly inhibited the proliferation, migration, and invasion of NSCLC cells, as well as promoted apoptosis. More importantly, silencing of PRDX2 could inhibit activation of the Akt/mTOR signaling pathway in NSCLC cells. Therefore, PRDX2 might serve as a potential therapeutic target for NSCLC treatment.

## Data Availability

All data generated or analyzed during this study are included in this published article.

## Abbreviations

NSCLC: Non-small cell lung cancer
PRDX2: Peroxiredoxin 2.
